# A Specific Carbohydrate Diet Virtual Teaching Kitchen Curriculum Promotes Knowledge and Confidence in Caregivers of Pediatric Patients with Inflammatory Bowel Disease

**DOI:** 10.3390/nu15183999

**Published:** 2023-09-15

**Authors:** Nancy Rivera, Kaylie Nguyen, Venus Kalami, Feifei Qin, Maya B. Mathur, Rebecca Blankenburg, Ann Ming Yeh

**Affiliations:** 1 Division of Pediatric Hospital Medicine, The Permanente Medical Group, Santa Clara, CA 95051, USA; 2 Stanford Children’s Health, Lucile Packard Children’s Hospital, Palo Alto, CA 94304, USA; kanguyen@stanfordchildrens.org (K.N.); vkalami@stanfordchildrens.org (V.K.); 3 Quantitative Sciences Unit, Stanford University, Palo Alto, CA 94304, USA; fqin@stanford.edu; 4 Quantitative Sciences Unit and Department of Pediatrics, Stanford University, Palo Alto, CA 94304, USA; mmathur@stanford.edu; 5 Division of Pediatric Hospital Medicine, Department of Pediatrics, Stanford School of Medicine, Palo Alto, CA 94304, USA; rblanke@stanford.edu; 6 Division of Pediatric Gastroenterology, Hepatology, and Nutrition, Department of Pediatrics, Stanford School of Medicine, Palo Alto, CA 94304, USA; annming@stanford.edu

**Keywords:** inflammatory bowel disease, specific carbohydrate diet, virtual teaching kitchen, nutrition education, food as medicine, food knowledge

## Abstract

Diet-based approaches such as the Specific Carbohydrate Diet (SCD) have proposed health benefits for patients with Inflammatory Bowel Disease (IBD). Despite its potential effectiveness, patients and caregivers identified barriers towards implementing the SCD, and a majority expressed interest in formal education surrounding the SCD. This study aimed to determine the impact of a virtual teaching kitchen curriculum on caregivers’ knowledge and perspectives on implementing the SCD. Inclusion criteria included pediatric patients with IBD aged 3–21 years and their caregivers. Participants should have fewer than 12 months of experience with the SCD or have no experience with the SCD but with an interest in learning it. Twenty-three caregivers took part in a 90-min virtual teaching kitchen curriculum and completed pre- and post-session surveys. Caregivers had statistically significant increases in total curriculum scores (*p* < 0.0001) as well as increases in all curricular elements post-curriculum teaching. Caregivers indicated that they plan to apply the newly acquired recipes and cooking concepts and appreciated the encouragement and support they received during the course. Curricular strengths identified included the innovative multimodal curriculum structure and professional and community support. IBD centers can use this pilot study to create or expand SCD and other nutritional curricula for the IBD community.

## 1. Introduction

Inflammatory Bowel Disease (IBD) is a chronic inflammatory condition that involves immune-mediated damage to the gastrointestinal tract. One-quarter of all IBD cases present before a patient is 20 years old. In a study based on data from 1996 to 2006, the incidence of pediatric IBD was 10 in 100,000 children in the United States and Canada [1]. This number is likely to have risen over the last decade, as pediatric centers globally have reported an increasing incidence of IBD in children [2,3].

IBD encompasses two main conditions: Crohn’s Disease (CD) and Ulcerative Colitis (UC). In the pathogenesis of pediatric IBD, there exists a complex interplay between genetics, the immune system, and the gut microbiome. Current treatments for IBD include corticosteroids, immunomodulators, and biologics, which largely target the immune dysregulation of the host. Studies have shown that IBD patients exhibit significant alterations in the gut microbiome as compared to controls for CD and UC in both adults and children [4,5]. For example, in a 2019 study by Kowalska-Duplaga et al., newly diagnosed pediatric CD patients had an abundance of the *Enterococcus* genus and a reduction in other beneficial flora such as *Bifidobacterium* (*B. adolescentis*), *Ruminococcus* (*R. bromii*), and *Faecalibacterium* (*F. prausnitzii*), among others [4]. This alteration or dysbiosis plays a key role in driving gut inflammation and thus disease processes and activity. 

Recent scientific advances have improved our understanding of the complex relationship between diet, the gut microbiome, and the pathophysiology and treatment of IBD. Although the pathophysiology of IBD is multifactorial, increasing evidence points to diet as an important factor that may increase the risk of developing IBD. Lack of a varied diet and fiber- and probiotic-rich foods, in addition to a diet more reliant on ultra-processed foods, excessive added sugars, certain preservatives, and emulsifiers, may have a detrimental effect on the development of a healthy digestive system [6] and gut flora. Dietary interventions are a potent way to specifically induce desired microbiota changes and ultimately decrease inflammation [7]. From a treatment standpoint, dietary therapy is an attractive alternative to medications as it may allow certain diet-responsive IBD patients to decrease their disease burden without additional immunosuppression. It is also viewed as more natural by families and clinicians and can offer a course of treatment that feels empowering to the patient and the family. Further, diet therapy may be used as an adjunct to medications, especially when patients may only have a partial disease response to immunosuppression. Various diet-based approaches, such as the Crohn’s Disease Exclusion Diet, Exclusive Enteral Nutrition, the Anti-Inflammatory Diet, and Specific Carbohydrate Diet (SCD), have been studied and found to be effective in treating diet-responsive patients with IBD [8,9]. 

One elimination diet studied is the SCD. This diet emphasizes whole, unprocessed foods such as most fruits and vegetables, nuts, seeds, and animal proteins. It also allows lactose-free dairy such as aged cheeses and butter as well as a 24-hour fermented SCD yogurt, which provides beneficial probiotics. The diet also emphasizes the inclusion of whole, nutrient-dense, unprocessed foods. It excludes food additives, all grains, most starches, and most sweeteners outside of honey and dates. The underlying rationale of the SCD is based on the idea that certain sugars and complex carbohydrates may not be adequately or completely digested and absorbed in the body. The byproduct of this incomplete digestion subsequently causes an imbalance or dysbiosis in the gut bacteria, which in turn causes intestinal inflammation and immune dysregulation [10]. Recent research from Wastyk et al. describes participants who ate fermented foods had increased microbiota diversity and decreased overall inflammation. Therefore, the inclusion of probiotics in the form of fermented foods such as SCD yogurt may provide similar benefits [11]. 

Research on the SCD has shown promising results in pediatric patients with IBD. A 12-week study by Suskind et al. investigated the SCD’s effect on children with both CD and UC [12]. Suskind’s team reported that the SCD led to clinical and biochemical improvements, normalized disease activity scores in both UC and CD, shifted the microbiota composition, and reduced inflammatory markers. However, the diet was not effective for two patients who could not maintain participation in the study and diet. A second study by the same group compared the SCD, a modified SCD, and a healthy whole foods diet in CD patients [13]. This study concluded that the SCD compared to the modified SCD had better outcomes for symptom relief and inflammation. A large multicenter study, PRODUCE, compared the SCD and a modified SCD using an n-of-1 research methodology [14]. Interestingly, the study showed that both diets had similar benefits over a standard diet in improving symptoms and calprotectin measures. This is promising as the modified SCD is more attainable for many families given the inclusion of starches such as rice and sweet potato. Though emerging research suggests that the SCD causes a shift in intestinal microbiota composition, no consistent change or effect has been elucidated in the limited research thus far [12]. A case study published in *JPGN Reports* in August 2023 by David Simon et al. described an 8-year-old female with perianal and ileocolonic CD who utilized the SCD as monotherapy for both induction and maintenance who not only showed gradual improvement over time clinically and based on laboratory evaluation but also demonstrated endoscopic, radiographic, and histologic remission by 1 year [15].

Due to the promising preliminary research on the SCD, patients and families have indicated significant interest in utilizing the SCD to treat IBD. A recent online international needs assessment survey of over 200 participants indicated high utilization of online resources and strong interest in a formal curriculum on the basics of implementing the SCD [16]. Formal training includes SCD courses or one-on-one instruction from a registered dietitian, physician, or advanced practice provider. The results of this survey indicated a gap in the SCD education available and the desire for patients and families to learn directly from healthcare professionals, rather than from online parent support groups. Prior studies on the SCD, such as the PRODUCE study [14], utilized one-on-one 60-min nutrition consultations with registered dietitians, phone follow-ups, and 30-min consultations at follow-up visits. This type of formal one-on-one education, while effective for knowledge transfer, is time-intensive and not available at every IBD center. The study displays the extent of support families need to implement the SCD successfully and shows how most diet and behavior changes require frequent and regular touchpoints to make lasting behavioral change. 

Culinary medicine is an emerging evidence-based interdisciplinary field that utilizes nutrition science, culinary arts, and medical education to teach patients how to treat and prevent diet-related disease [17]. Teaching kitchens are used in culinary medicine to improve both nutritional knowledge and hands-on cooking skills. Teaching kitchens are a unique way to allow for direct interaction with the provider and the learner to enhance skills and increase nutritional knowledge and efficacy. Teaching kitchens have been shown to be well-received, financially feasible, and effective [17]. Recent reports of electronic consultations or virtual interventions utilizing culinary medicine principles have also improved accessibility and were reimbursable by insurance [18]. Teaching kitchens have been shown to improve dietary patterns in chronic medical conditions such as diabetes, cardiovascular disease, and metabolic syndrome [19,20]. Participants of teaching kitchen interventions were more likely to adopt healthier eating patterns, increase consumption of fruits and vegetables, and have improved confidence in reading nutritional labels [19]. 

The SCD is restrictive in nature and does not allow for many processed or pre-packaged foods. Patients or caregivers of patients on the SCD therefore need to have baseline culinary skills, be adept at reading and interpreting nutrition labels, and be ready to adapt recipes to SCD guidelines. Initiation of the SCD presents a steep and ongoing learning curve [16] and can be a challenge for many families given taste preferences, cost, increased cooking time, and having family member buy-in. Therefore, we hypothesized that the incorporation of culinary medicine principles and hands-on instruction in a teaching kitchen would be an advantageous way to teach patients or caregivers the SCD. Given the need for reputable curricular resources to teach the SCD and the desire from the community for an interactive and hands-on curriculum, this study aimed to determine the impact of a virtual teaching kitchen curriculum on caregivers’ knowledge and perspectives of cooking the SCD for pediatric patients with IBD. This manuscript formally describes the virtual teaching kitchen curriculum and evaluates its impact on caregivers’ knowledge and perspectives.

## 2. Materials and Methods

### 2.1. Participants

Participants were recruited via flyers distributed virtually and in a clinic. Recruitment flyers were posted at the Stanford Children’s Pediatric Gastrointestinal Clinic and the Stanford Children’s Pediatric Integrative Medicine Clinic. Virtual recruitment flyers and recruitment emails were shared via an internal secure patient listserv, local pediatric gastrointestinal clinics, the Facebook *SCD Families* support group (https://www.facebook.com/groups/SCDFamilies, accessed on 15 January 2021, IBD nutrition-oriented websites (nimbal.org, accessed on 15 January 2021), and a nonprofit online resource for nutrition therapies for IBD (www.NTforIBD.org, accessed on 15 January 2021. All interested participants were screened for eligibility by telephone or email. Eligible participants completed informed consent and assent forms using Qualtrics Software. Inclusion criteria included pediatric patients with IBD aged 3–21 years and their caregivers, having less than 12 months of experience with the SCD or having no experience with the SCD but having an interest in learning about the SCD.

### 2.2. Study Design

The intervention was guided by the self-determination theory [21] and the social cognitive theory [22]. The self-determination theory suggests that an individual’s motivation is based on the triad of competence, autonomy, and relatedness: competence to be able to master a topic, autonomy to have the choice regarding a behavior, and relatedness to feel a sense of belonging. Social cognitive theory is centered around the dynamic relationship between cognitive (knowledge and skill to complete a behavior), behavioral (self-efficacy), and environmental (observational learning) factors.

Online, de-identified pre- and post-intervention surveys obtained the demographic information of the caregiver and the clinical characteristics of the pediatric patient with IBD and explored the caregiver’s knowledge and perspectives surrounding the SCD as well as future directions for SCD education. The pre- and post-intervention surveys were completed by the caregiver and were matched for data analysis via a personal survey code (Supplementary Table S1).

### 2.3. Curriculum

The online teaching kitchen curriculum was developed by a team of pediatric healthcare professionals (pediatricians, pediatric gastroenterologist, nurse practitioner, dietitian nutritionist, and medical educators) using Kern’s six steps of curriculum development [5]. The educational content and multimodal curriculum were based on results from an SCD Needs Assessment, which explored the perspectives of 208 caregivers on SCD education [16]. 

Three virtual sessions were held based on participant availability. Each caregiver took part in one 90-min online Zoom session. It was voluntary for the pediatric patient to attend the session. The course was initially intended to be in-person with a hands-on teaching kitchen; however, given COVID-19 pandemic restrictions, the curriculum was transitioned from in-person to a virtual teaching kitchen. The curriculum was named SCRUMPTIOUS (Specific Carbohydrate Diet Curriculum—Patient and Caretakers Outlooks) and included live and pre-recorded didactic content. 

Teaching modalities for the curriculum included a combination of didactics, small group discussions, interactive polls, and educational videos, including virtual teaching kitchen videos. The pre-recorded didactic content of the curriculum included the following topics: legal vs illegal SCD foods, nutritional adequacy of the SCD, select SCD recipes, and the science behind the SCD. The virtual teaching kitchen videos of select SCD recipes were also pre-recorded at a professional kitchen space with a hired videographer service. Live sessions included the introduction, word cloud activity, question and answer (Q&A), and wrap-up sessions. All four presenters participated in all the live sessions. The introduction section encouraged participants to introduce their name, their role as a caregiver, the patient with IBD, and their experience with IBD and the SCD. The word cloud question included: “What do you think of when you hear the term SCD”. The question and answer sessions were moderated by one presenter who read questions asked in the chat aloud. Participants were encouraged to turn on their video cameras to optimize opportunities for interaction and engagement during these live sessions. 

All curriculum content was recorded and uploaded online to become evergreen content for reference by participants and to be easily accessed by future learners and the public. Both Vimeo and YouTube were used as the platforms for the videos. Curriculum link: https://www.stanfordchildrens.org/en/service/inflammatory-bowel-disease/services/scrumptious.

The curriculum timeline (with materials) was as follows:
90 min Lesson Plan (Supplementary Table S2):
0–10 min: Introductions, Word Cloud, Pre-Survey(Real Time)10–23 min: SCD Primer Video(Pre-Recorded)23–33 min: Nutritional Adequacy Video(Pre-Recorded)33–36 min: Q&A from chat(Real Time)36–50 min: Three Recipe Videos(Pre-Recorded)50–53 min: Q&A from chat(Real Time)53–69 min: The Science Behind SCD Video(Pre-Recorded)69–76 min: How to Make Dairy Yogurt Video(Pre-Recorded)76–81 min: Yogurt Quiz(Real Time)81–90 min: Reflections, Word Cloud, Post-Survey(Real Time)

### 2.4. Statistical Analysis 

We analyzed all participants who completed both the pre- and post-session surveys, and the baseline demographic and clinical characteristics of the cohort were described using frequencies with percentages for categorical variables. Curriculum outcomes were measured on a 4-point Likert scale ranging from ‘strongly disagree’ to ‘strongly agree’, and responses were dichotomized as ‘strongly agree/agree’ vs. ‘strongly disagree/disagree’. We presented frequency counts and calculated means along with standard deviations for each outcome pre- and post-curriculum. Additionally, we created a composite outcome score by summing up the score for each of the outcome measures and compared the composite value pre- and post-curriculum using a paired *t*-test. All statistical tests were evaluated at an alpha level of 0.05. Analyses were conducted using SAS 9.4 (SAS Institute Inc., Cary, NC, USA) and R version 4.0 (R Core Team, Vienna, Austria).

Content analysis was used to analyze the qualitative data. Two investigators (N.R. and K.N.) reviewed the data independently, assigned codes, and then compared with each other until a consensus was achieved. Additional investigators (A.M.Y. and V.K.) helped reconcile any conflicts. Frequency counts were determined by the number of times a code was mentioned.

The SCRUMPTIOUS website analytics were run by the Stanford Children’s Health web team and Google^TM^ Looker Studio (Google, Mountain View, CA, USA) to calculate engagement metrics for the SCRUMPTIOUS website and YouTube and Vimeo videos from 1 June 2021 to 31 August 2023.

The study was approved by the Institutional Review Board (IRB) of Stanford University (Protocol Number: 55032).

## 3. Results

### 3.1. Patient Demographics

After screening 34 participants, 31 participants were determined to be eligible for the study. In total, 25 caregivers consented, and 23 caregivers completed the study, with a retention rate of 92%.

Caregivers were defined as the parent/caretaker of the individual with IBD, and 60.9% were female (Table 1). Most caregivers, 82.6%, resided in the United States, while 17.4% were from an international country. Approximately 47.8% of the caregivers initiated the SCD for the pediatric patient with IBD to avoid medication, while 26.1% used the SCD as an adjunct to medication therapy. The majority, or 65.2%, of caregivers had never received any formal training on the SCD. Of the pediatric patients with IBD, 56.5% were 0–10 years of age at diagnosis, 60.9% had Crohn’s Disease, and 78.3% were actively following the SCD, with 82.3% of those individuals following a strict SCD and only modifying it less than five percent of the time.

### 3.2. Curriculum Outcomes

Table 2 displays the outcome scores reported by the caregivers, both pre- and post-curriculum. All aspects of knowledge and perspectives improved after the curriculum, with the curriculum composite outcome score being significantly higher post-curriculum compared to pre-curriculum, with a mean difference in score of 5.17 out of a total of 40 points (95% CI: 3.48–6.87; *p*-value <0.0001) (Table 3). Regarding knowledge outcomes, caregivers’ scores improved on the following:

- Understanding legal (allowed) versus illegal (not allowed) foods on the SCD;
- Liberalization of the SCD, or when and how to add back foods that were not allowed on the strict version of the SCD;
- Maintaining a balanced diet on the SCD, determining nutritional adequacy, and potential need for nutritional supplements such as calcium, iron, zinc, and multivitamins when there may not be nutritional adequacy in the diet;
- Comprehension of the science as well as gaps in scientific knowledge about the SCD;
- Concepts of meal preparation, substitution of ingredients to make a recipe SCD-friendly, in addition to meal and snack ideas.

**Figure 1:**
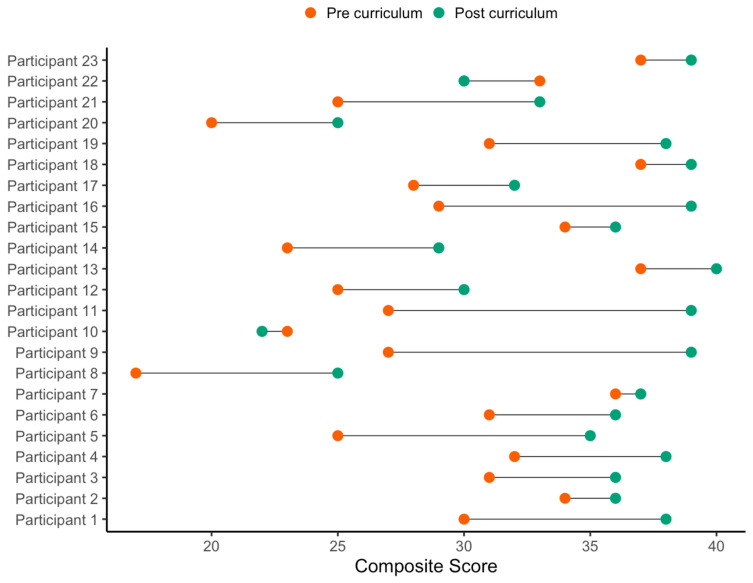
Pre- and Post-Curriculum Composite Scores by Participant.

Regarding perspectives, caregivers’ scores improved on confidence and motivation to prepare SCD meals and snacks. The acknowledgement of barriers also increased post-curriculum. Figure 1 displays the pre- and post-curriculum composite scores by participant. Out of the 23 participants, 21 participants had higher post-curriculum composite scores. Participants 10 and 22 showed reduced post-curriculum composite scores by two and three points, respectively.

Participants reported various aspects that they learned and would apply to SCD cooking because of the curriculum: 43% of participants learned new recipes and cooking concepts, 20% expressed gratitude for support and encouragement, 17% gained knowledge about nutritional adequacy, and 14% could clarify the distinction between legal vs illegal foods. Barriers to implementing what participants learned included the time and burnout associated with the SCD (32%), having children who are picky eaters (18%), the limited variety of foods available (18%), fulfilling the needs of the entire family (14%), and the steep learning curve (11%) (Table 4). Curriculum strengths in order of preference included curriculum structure, professional support, community support, topics such as the science behind the SCD, and cooking videos (Table 5). Participants indicated that they would benefit from more personalized sessions, curricula with more sessions, more kid-friendly content/involvement, and more peer-to-peer interactions as potential ways to improve future curricula. For additional topics of interests for SCD cooking videos, see Supplementary Table S3. 

### 3.3. Website

A total of 2818 users accessed the SCRUMPTIOUS website directly, over 3564 different sessions. Of those users, 25.6% were returning users. The majority of users, or 55.9%, viewed the SCRUMPTIOUS website via a mobile device. 

The SCRUMPTIOUS videos were viewed a total of 6555 times. When looking at the videos themselves, the Primer on SCD full-length video that was about 40 min long had the most views at 3763 views, with an average of 27% of the video length watched. Overall, the videos related to the science of the SCD had the most views, with a range of 325 to 3763. The cooking videos had a range of views from 42 to 302, with 42.5% of the video watched on average. The average viewing duration for the cooking videos ranged from 1 min and 14 s to 2 min and 44 s.

## 4. Discussion

This is the first published virtual teaching kitchen curriculum developed to educate, motivate, and support pediatric patients and their caregivers as they begin the SCD. We found a statistically significant improvement in overall participant knowledge and positive perspectives about the SCD after completion of the curriculum. Specifically, many caregivers expressed appreciation for the emotional, communal, and educational support from IBD and SCD peers. They valued the multimodal curriculum structure that incorporated real-time, evidence-based discussion with professionals in the field. Participants also identified topics of interest and curriculum strengths that future curricula can leverage from and build upon. 

This is the first formalized and real-time SCD virtual teaching kitchen curriculum widely available for patients and families with IBD. While other excellent online resources exist, they are commonly pre-recorded or in written form, with very limited access to real-time discussion. The uniqueness of the curriculum was its survey-informed structure, in which patients with IBD who followed the SCD and their caregivers guided the curriculum content and structure [16]. Other advantages of the curriculum are that healthcare professionals led virtual, real-time, patient-centered interactive discussions and incorporated a virtual teaching kitchen in the form of pre-recorded cooking videos preparing SCD foods. Participants had the opportunity to ask questions after each video. They appreciated the community aspect of the curriculum and found the support and encouragement from the speakers as well as fellow participants to be beneficial. Additionally, the multimodal structure appeals to individuals with different learning styles. Further, the qualitative feedback for improvement mostly focused on requests for personalized sessions, sessions with peer-to-peer interaction, or more involvement of the children.

In the current era, patients often turn to social media platforms for medical information, which is not always reliable or research-based. Offering unbiased, research-backed resources from medical experts is crucial for advancing medical and nutritional education for IBD patients. The data from YouTube and Vimeo showed that the science-related content about the SCD was the most viewed, as compared to the cooking videos. Out of all the videos, the cooking videos were viewed the least, with an average viewing duration between one and two minutes. Though the qualitative feedback did not specifically comment on the cooking video length, based on the viewing data, we surmise that providing brief cooking videos to mimic videos that are currently popular on social media platforms such as TikTok and Instagram might encourage more views. 

Despite initially developing the curriculum as an in-person teaching kitchen, the COVID-19 pandemic required conversion of the curriculum to an online teaching kitchen format. This compulsory pivot allowed the curriculum to reach a wider audience and enabled access to the curriculum for SCD users worldwide. Furthermore, the pre-recorded cooking videos and lectures that were interspersed between live discussions allowed for participants to rewatch, fast forward, and even share with family members and caregivers of each patient. 

It is important to acknowledge the limitations of the study. First, the study had a risk of selection bias. The curriculum was led in English, it was online, and many of the featured recipes were catered to a North American audience, which collectively limited linguistic inclusivity and cultural relevance. Time zone limitations and Internet accessibility may have created further access barriers. Moreover, there is a risk of selection bias as the recruitment flyers were shared on IBD nutrition-oriented websites nimbal.org and NTforIBD.org as well the social media group *SCD Families* on Facebook (https://www.facebook.com/groups/SCDFamilies) where many families already had knowledge about the SCD. It is crucial to acknowledge that this study was conducted on caregivers of patients with varying levels of prior exposure to SCD to increase enrollment into the course. However, if the study was solely on patient families completely new to the SCD, the observed effect might have been more pronounced. We also need to point out that 2 of the 23 participants had slightly reduced post-curriculum composite scores. Clearly, the curriculum may not benefit everyone given the wide range in experiences with SCD, ages of the children on the SCD, lifestyles, backgrounds, food preferences, and learning styles amongst the participants. Furthermore, the course took place during a time when many schools and workplaces were mandated to work via video. Therefore, it is also possible that some participants might have experienced video conferencing burnout. When looking at the study population, most participants were female caregivers who were primarily responsible for shopping and cooking in the household. It is possible that parents and caregivers who are not responsible for household food decisions were not attracted to the study or able to participate. Additionally, the SCD requires significant time investment, effort, and financial commitment to be implemented successfully, and families with limited access to these resources may not be represented in the study. A further limitation is that the curriculum focuses solely on the SCD and thus inherently excludes learners who are utilizing other therapeutic diets to manage their IBD. 

Our main takeaways from this pilot teaching kitchen curriculum for SCD include the following:

- The importance of community building to foster motivation and engagement at the start and end of the session.
- The multimodal curriculum structure that combined live interactive sessions with short, pre-recorded videos worked well.
- The recorded sessions of the didactic and teaching kitchen segments were valuable as evergreen content.
- Participants valued live participation segments and requested more live and interactive sessions in the future.

In forthcoming SCD education projects, this curriculum can be modified to focus on training healthcare professionals on how to teach the content. Teaching kitchens to “train the trainer” have been successful in improving healthcare provider attitudes, knowledge, and behaviors regarding healthy food choices [23]. A similar approach could be utilized to educate professionals about diets such as the SCD at institutions where registered dietitians, advanced practice providers, or physicians with specific expertise may be limited. This would help to improve access to clinicians who are knowledgeable about therapeutic diets for IBD. Similar teaching kitchen curricula can further be utilized in other pediatric GI conditions requiring specialized diets such as eosinophilic esophagitis or celiac disease. 

There are many future directions to improve and personalize this curriculum to meet individual needs. For one, the sense of community can be improved by implementing hands-on teaching kitchens as opposed to virtual formats. Additionally, small group sessions could be adapted to include child-friendly cooking techniques and SCD-friendly recipes for pediatric patients and their families. Obtaining feedback on patient satisfaction would also further complement caregiver responses. Future studies would benefit from measuring behaviors prior to and after SCD education to assess the curriculum’s impact on food-related behavior change, as opposed to relying on participant reports on intended change. The curriculum was tailored to an audience with fewer than 12 months of experience on the SCD, which constitutes a large variation in knowledge and motivation. Forthcoming studies can adapt the curriculum content more precisely to the degree of experience of the learner, which would likely improve participant engagement and satisfaction with the curriculum. To address a more diverse and inclusive audience, subsequent educational projects should address the unique language and cultural needs of the patients and caregivers, as well as populations of lower social economic status.

## 5. Conclusions

The SCRUMPTIOUS virtual teaching kitchen curriculum provided an innovative multimodal educational experience for patients with IBD who expressed interest in diet-based therapies for IBD management. This curriculum was informed by a thorough needs assessment survey, which shaped the curriculum structure, topics, and priorities, ensuring that the curriculum content matched the IBD community’s needs and interests [16]. The curriculum was met positively by attendees worldwide, and post-curriculum surveys revealed that SCD-related knowledge, confidence, motivation, and skills significantly improved amongst caregivers of patients with IBD. This pilot study serves as a strong framework for other IBD centers to build coordinated SCD and IBD nutrition curricula for the IBD community to help increase access to IBD diet materials while increasing IBD diet knowledge and confidence and fostering community amongst pediatric IBD families. Future research on IBD diets can focus on personalized education by aligning lesson plans with participant skill and experience level, possibly recruiting newly diagnosed patients or caregivers with no prior knowledge of the SCD. More comprehensive engagement could be achieved by enhancing accessibility, addressing diverse linguistic and cultural needs, and involving additional family members. The SCRUMPTIOUS virtual teaching curriculum has demonstrated the need for and communal appreciation of this clinical educational approach which other centers, clinics, and organizations can adopt and modify for their unique community needs. 

## Figures and Tables

**Table 1:** Demographics and Clinical Characteristics of Participants.

Caretaker Demographics, *n* = 23			
Caretaker Sex	*n* (%)	Residence	*n* (%)
Female	14 (60.9)	California	9 (39.1)
Male	8 (34.8)	Rest of the United States	10 (43.5)
Prefer not to say	1 (4.3)	International	4 (17.4)
**Main reason to consider or to start the SCD initially *n* (%)**			
To avoid medication therapy			11 (47.8)
To add as a supplement to medication therapy			6 (26.1)
To try an integrative medicine approach			3 (13.0)
Recommended by medical provider			1 (4.3)
Recommended by family member or friend			1 (4.3)
Other			1 (4.3)
**Received any of the following training(s) on the SCD? ^a^**			***n* (%)**
Never			15 (65.2)
A session with a medical provider on the SCD			5 (21.7)
A session with a registered dietitian on the SCD			10 (43.5)
Attended a lecture or seminar on the SCD			1 (4.3)
Attended a teaching kitchen on the SCD			0 (0.0)
**Clinical Characteristics of Pediatric Patients with IBD, *n* = 23**			
**IBD diagnosis age**	***n* (%)**		
0–10 years old	13 (56.5)		
11–17 years old	9 (39.1)		
18–21 years old	1 (4.3)		
**Type of IBD**		***n* (% of cohort)**	***n* (% of disease)**
Crohn’s Disease		14 (60.9)	
Esophagus and Stomach Small Bowel (duodenum, jejunum, ileum) Large Bowel (colon) Extraintestinal (skin, joint, other) Unsure			0 (0) 6 (42.8) 6 (42.8) 1 (7.1) 1 (7.1)
Ulcerative Colitis Large Bowel (colon) Unsure		4 (17.4)	4 (100) 0 (0)
Indeterminate Colitis Esophagus and Stomach Small Bowel (duodenum, jejunum, ileum) Large Bowel (colon) Extra intestinal (skin, joint, other) Unsure		2 (8)	0 (0) 0 (0) 4 (100) 0 (0) 0 (0)
Unsure		1 (4.3)	
Unsure			1 (100)
**Individual with IBD following SCD now?**	***n*** **(%)**	**Degree that individual with IBD follows SCD, *n* = 17**	***n*** **(%)**
Yes	17 (78.3)	Strict SCD (<5% modifications used)	14 (82.4)
No	6 (21.7)	Minor modifications (modify < 25% of the time)	1 (5.9)
Unsure	0 (0.0)	Major modifications (modify > 50% of the time)	1 (5.9)

^a^ Participants could have received more than one type of training.

**Table 2:** Caregiver Knowledge and Perspectives Outcome Scores from Pre- and Post-Curriculum.

Outcome	Pre-Curriculum *n* = 23	Post-Curriculum *n* = 23
	Mean (SD)	Mean (SD)
**I know how to determine legal vs illegal SCD foods**	3.17 (0.65)	3.57 (0.66)
**I understand how to liberalize SCD**	2.09 (0.95)	3.22 (0.74)
**I understand what nutritional supplements may be needed for my child while on SCD**	2.09 (0.85)	3.09 (0.67)
**I understand the science behind how SCD works for IBD**	2.87 (0.69)	3.70 (0.47)
**I feel confident preparing SCD yogurt**	3.13 (1.14)	3.65 (0.71)
**I feel motivated to prepare SCD-legal meals/snacks**	3.70 (0.47)	3.74 (0.45)
**I feel confident in preparing SCD-legal meals/snacks**	3.13 (0.87)	3.57 (0.66)
**I feel knowledgeable about preparing SCD-legal meals/snacks**	3.00 (0.95)	3.48 (0.67)
**I have the skills necessary to prepare SCD-legal meals/snacks**	3.35 (0.71)	3.57 (0.73)
**I face barriers when it comes to preparing SCD-legal meals/snacks**	2.70 (0.82)	2.83 (0.78)

**Table 3:** Caregiver Composite Outcome Scores from Pre- and Post-Curriculum.

	Pre-Curriculum *n* = 23	Post-Curriculum *n* = 23
	Mean (SD)	Mean (SD)
**Composite outcome score**	29.22 (5.57)	34.39 (5.22)
**T-test of paired differences (post/pre)**	**Mean (95% CI)**	
	5.17 (3.48, 6.87) *p*-value < 0.0001	

**Table 4:** Barriers to Implementing What Was Learned as Identified by Caregivers, *n* = 23.

Theme	% Response by Participants	Associated Quotes
**Time and Burnout**	32%	“SCD is very time consuming. When we are back to all our activities after COVID, it will feel overwhelming to do this level of food prep” “Fatigue. Honestly, we’re cooking like we live on a farm and we live in the suburbs. It’s awesome and we feel great. But truly my legs hurt from standing in the kitchen so much”
**Picky Eating**	18%	“A child who is a picky eater and a vegetarian”
**Limited Variety of Food Available**	18%	“Feeling guilty about a restricted diet—And knowing how much “easier” modified SCD would be” “Menu planning and keeping a balance and variety with my son’s taste buds”
**Fulfilling the Needs of the Entire Family**	14%	“Having a 9 year old and 5 other kids in our home, it will be hard to prepare foods similar to things he loves and his siblings eat.”
**Steep Learning Curve**	11%	“Time for food prep, planning ahead, etc., is the biggest barrier, but the more information I learn, the easier this is to do”

**Table 5:** Curriculum Strengths Identified by Caregivers, *n* = 23.

Theme	% Response by Participants	Associated Quotes
**Curriculum Structure**	26%	“Nice transitions from one topic to the next…fun to watch, very positive and supportive atmosphere. The practitioners were all very knowledgeable and did a fantastic job answering all the questions that came up” “I liked the interactive chats, the recipe videos, the scientific information about the efficacy”
**Professional Support**	26%	“I really appreciated the wealth of information shared, and most especially the chance to ask questions after the information was presented, with both knowledgeable and kind professionals as well as other families, to share from their experiences” “Doctors explaining the answers to our questions”
**Community Support**	17%	“Seeing other families on SCD” “Great energy from everyone, great professionals, nice to see other families dealing with same things” “Talking to all of you and hearing from you. It’s a lonely road”
**Learning the Science**	15%	“The science behind SCD and how amazing [and] genuine the presenters were to help the audience” “I liked [to] hear … about the science behind the diet...I liked hearing why it works and what is happening with the gut with IBD”
**Cooking Videos**	15%	“The cooking lessons were fun, always helpful to get new recipe ideas” “As it was, the recipe videos did serve to give people in the class a chance to see a recipe done and think ‘I could do that’”

## Data Availability

The data presented in this study are available on request from the corresponding author. The data are not publicly available in accordance with study consent provided to participants.

## Supplementary Materials

The following supporting information can be downloaded at: https://www.mdpi.com/article/10.3390/nu15183999/s1, Table S1: Pre- and Post-survey exploring knowledge and perspectives, Table S2: Curriculum Lesson Plan, Table S3: Additional Topics for future SCD-Cooking Videos.
