# Supplementary material for: A Specific Carbohydrate Diet Virtual Teaching Kitchen Curriculum Promotes Knowledge and Confidence in Caregivers of Pediatric Patients with Inflammatory Bowel Disease
Source: Nutrients. 2023 Sep 15;15(18):3999. doi: 10.3390/nu15183999 (PMC10537188; doi:10.3390/nu15183999)
Supplement: Supplementary file 1 [file nutrients-15-03999-s001.zip › nutrients-2598589-supplementary.pdf]

SUPPLEMENTAL TABLE 1 (Table S1): Pre- and Post-survey exploring knowledge and perspectives

	Strongly Disagree	Disagree	Agree	Strongly Agree
I know how to determine legal vs illegal SCD foods	1	2	3	4
I understand how to liberalize SCD	1	2	3	4
I understand what nutritional supplements may be needed for my child while on SCD	1	2	3	4
I understand the science behind how SCD works for IBD	1	2	3	4
I feel **confident** preparing SCD yogurt	1	2	3	4
I feel **motivated** to prepare SCD- legal meals/snacks	1	2	3	4
I feel c**onfident** in preparing SCD-legal meals/snacks	1	2	3	4
I feel **knowledgeable** about preparing SCD-legal meals/snacks	1	2	3	4
I have the **skills** necessary to prepare SCD legal meals/snacks	1	2	3	4
I face **barriers** when it comes to preparing SCD- legal meals/snacks	1	2	3	4

SUPPLEMENTAL TABLE 2 (Table S2): Curriculum Lesson Plan

Amount of Time	Tools	Learning Activity	Presenter
(10 min)	*Add pre-survey link to chat<br>*Slides for intro<br>*Chat for word cloud	Pre-Survey<br>Introduction<br>Word Cloud	"insert name"
(13 min)	*SCD primer video	SCD PRIMER	
(10 min)	*Nutrition Adequacy Video	Nutritional Adequacy	
(3 min)	Q&A from chat	Q&A/ Break	
(14 min)	Cracker Video (4m)<br>Muffin Video (6m)<br>Sausage Video (4m)	Recipe Videos	
(3 min)	Q&A from chat	Q&A/ Break	
(16 min)	*The Science Behind SCD video	Science behind SCD	
(7 min)	*Dairy Video	Yogurt Video	
(5 min)	Quiz Slide	Yogurt Quiz	
(9 min)	*Add post-survey link to chat<br>*Slides for resources<br>*Chat for word cloud	Post- survey<br>Reflections<br>Word cloud	

SUPPLEMENTAL TABLE 3 (Table S3)- Additional topics for future SCD-cooking videos

Kid friendly
Easy to make
On the go
Allergen specific (ex Nut free or Dairy free)
Breakfast
Family style dinners
Snacks
Smoothies
Meal planning
Eating Out
Travel
Where to find SCD legal ingredients
Organization of SCD ingredients
